# Effect of Xiaoyaosan Decoction on Learning and Memory Deficit in Rats Induced by Chronic Immobilization Stress

**DOI:** 10.1155/2013/297154

**Published:** 2013-12-29

**Authors:** Zhen-Zhi Meng, Jia-Xu Chen, You-Ming Jiang, Han-Ting Zhang

**Affiliations:** ^1^School of Preclinical Medicine, Beijing University of Chinese Medicine, No. 11, Beisanhuan Donglu, Chaoyang, Beijing 100029, China; ^2^School of Preclinical Medicine, Youjiang Medical University for Nationalities, Baise, Guangxi 533000, China; ^3^Department of Basic Theory in Chinese Medicine, Henan University of Traditional Chinese Medicine, Zhengzhou 450008, China; ^4^Department of Behavioral Medicine & Psychiatry, West Virginia University Health Sciences Center, Morgantown, WV 26506-9137, USA

## Abstract

Xiaoyaosan (XYS) decoction is a famous prescription which can protect nervous system from stress and treat liver stagnation and spleen deficiency syndrome (LSSDS). In this experiment, we observed the effect of XYS decoction on chronic immobilization stress (CIS) induced learning and memory deficit in rats from behaviors and changes of proteins in hippocampus. We used XYS decoction to treat CIS induced learning and memory deficit in rats with rolipram as positive control, used change of body weight and behavioral tests to determine whether the rats have LSSDS and have learning and memory deficit or not. We used Western blotting to determine the content of postsynaptic density protein 95 (PSD-95) and synaptophysin (SYP) in hippocampus. Results showed that XYS could improve the situation of slow weight gain induced by CIS, improve the ability of learning and memory, reverse the symptom of liver stagnation and spleen deficiency syndrome (LSSDS) in rats, and increase the levels of PSD-95 and SYP on the hippocampal nerve synapses. These findings suggested that XYS decoction may be helpful in reversing CIS induced learning and memory deficit by increasing the levels of PSD-95 and SYP on the hippocampal nerve synapses and improving synaptic plasticity.

## 1. Introduction

Learning and memory deficit is a very important symptom in central nervous system injury [1, 2]. Hippocampal synaptic plasticity plays a key role in learning and memory [3]. Hippocampus is a target of stress [4]. CIS significantly decreases the hippocampal volume by 3%–6.3% [5, 6], attenuates long-term potentiation (LTP) in hippocampal slices [7], and causes hippocampal CA3 apical dendritic retraction which parallels spatial memory impairments in male rats [8]. CIS causes dendritic atrophy of the hippocampal pyramidal neurons and alterations in the levels of neurotransmitters in the hippocampus [9]. Research found 602 genes in the hippocampus were differentially expressed in CIS (3 h per day for a 7-day period) induced rats, as well as 566 genes in hippocampus were differentially expressed in CIS (3 h per day for a 21-day period) induced rats [10]. CIS can not only induce spatial learning and memory deficit [11], but also result in rats with liver stagnation and spleen deficiency syndrome (LSSDS) in traditional Chinese medicine [12].

Traditional Chinese medicine (TCM) has an active effect on chronic disease and psychiatry. XYS decoction which was created in Song Dynasty (960-1127 AD) contains *Radix Angelicae Sinensis, Poria, Radix Paeoniae Alba, Radix Glycyrrhizae, Radix Bupleuri, Rhizoma Atractylodis Macrocephalae, Herba Menthae, and Rhizoma Zingiberis Recens*. The chemical constituent of XYS includes peoniflorin, saikoside, ferulic acid, atractylol, glycyrrhetate, curcumin, and menthone [13]. XYS decoction has been mainly used to treat LSSDS and mental disorders in TCM clinic [14, 15]. The function of XYS decoction is to soothe the liver, improve the circulation of qi, relieve depression, strengthen the spleen, and nourish blood. It is a safe and useful prescription in clinic. XYS decoction can protect nervous system from stress [16] and play a good role in the treatment of LSSDS [15, 17].

Our recently studies showed the XYS decoction reverse CIS-induced decreases in brain-derived neurotropic factor (BDNF) and increases tyrosine hydroxylase (TrkB) and neurotropic 3 (NT-3) in the frontal cortex and the hippocampal CA1 subregion [18]. The XYS decoction can significantly downregulate the contents of leptin and leptin receptor (*ob-R*) at the arcuate nucleus (ARC) in the basal of hypothalamus of chronic stressed rats [19]. Meanwhile, the XYS decoction containing serum significantly improves mitochondrial membrane potential and apoptotic rate of hippocampus neuron induced by oxidative stress [20].

The purpose of this study was to observe the effect of XYS decoction on chronic immobilization stress-induced learning and memory deficit in rats from behaviors and changes of proteins in hippocampus.

## 2. Materials and Method

### 2.1. Animal

Adult male Sprague-Dawley rats (Harlan, Indianapolis, IN.) weighing 227.2 ± 3.6 g (mean ± SD) were used for the experiments. Animals were housed in a temperature controlled room (22 ± 2°C) with a 12-h on/12-h off light cycle (lights on from 06:00 to 18:00). Water and food were freely available. All experiments were carried out according to the NIH Guide for the Care and Use of Laboratory Animals (NIH Publications No. 80-23, revised 1996). The procedures were approved by the Animal Care and Use Committees of West Virginia University Health Sciences Center.

### 2.2. Preparation of Extracts of the XYS Decoction

The XYS decoction consists of the following dried raw materials: 150 g of *Poria cocos* (Schw.) Wolf (Poria), 300 g of *Paeonia lactiflora *Pall. (Radix Paeoniae Alba), 150 g of *Glycyrrhiza uralensis* Fisch. (Radix Glycyrrhizae), 300 g of *Bupleurum chinense* DC. (Radix Bupleuri), 300 g of *Angelica sinensis* (Oliv.) Diels (Radix Angelicae Sinensis), 300 g of *Atractylodes macrocephala* Koidz. (Rhizoma Atractylodis macrocephalae), 100 g of *Mentha haplocalyx* Briq. (Herba Menthae), and 100 g of *Zingiber officinale* Rosc. (Rhizoma Zingiberis Recens). These eight herbs were purchased from Medicinal Materials Company of Beijing Tongrentang, processed in Sino-Japan Friendship Hospital (Beijing). We process herbs abiding by Regulation on Processing of Traditional Chinese Medical Herbal Pieces of Beijing.

### 2.3. Drugs and Treatments

XYS decoction (3.854 g/kg/d) was mixed by XYS powder and saline. Saline was mixed by NaCl (purchased from Fisher Scientific) and deionized water. The volume for XYS decoction or saline (i.g.) is 1 mL/100 g body weight. Rolipram was purchased from A. G. Scientific and was dissolved in saline containing 5% dimethyl sulfoxide (DMSO). The injection (i.p.) volume was 1 mL/kg body weight. Rolipram (0.3 mg/mL) was given once daily through the whole experiment.

Rats were divided into 4 groups, that is, control group, CIS + saline group, CIS + rolipram group, and CIS + XYS group. Every group had 10 rats. All groups, except control group, were immobilized for 3 hours per day through the whole experiment [11, 21, 22]. Saline or rolipram or XYS was given 30 min prior to CIS in the whole experiment. On 21st day, behavior tests such as locomotor activity, object recognition, or Morris water maze were taken after stress.

### 2.4. CIS Procedure

All groups, except the control group, were exposed to CIS. CIS was performed by putting rats in a breathable plastic decapicones for 3 h per day in the whole experiment until rats were sacrificed. It took first 21 days in the experiment to impair the ability of learning and memory and induce symptom of LSSDS in rats. The control group had access to food and water freely; however, food and water were not provided during stress.

### 2.5. Body Weight Monitoring and Behavioral Test Procedures

Body weight and general state were recorded before treatments. On day 21, behavior tests such as locomotor activity, object recognition, or Morris water maze were performed after stress. Rats were placed in the testing room 30 min before the behavioral tests for habituation. All the behavioral data were recorded manually by an unbiased observer.

#### 2.5.1. Locomotor Activity Test

**Figure 1:**
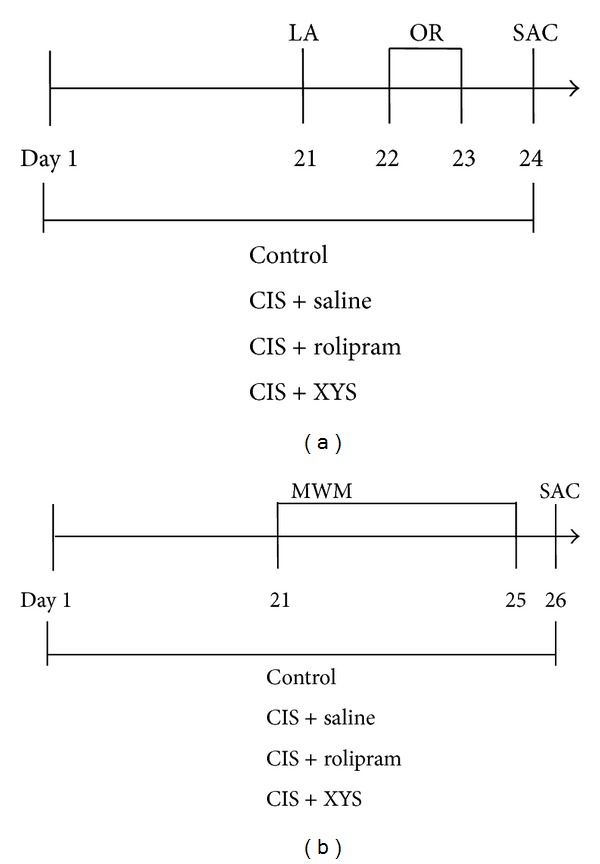
Schedule of drug treatments and behavioral tests. Saline and XYS were taken (i.g.) and rolipram (0.3 mg/kg) was injected (i.p.) once daily through the whole experiment. CIS was given 30 min after saline, XYS, or rolipram. Behavioral tests were carried out on day 21 (after the CIS). After all behavioral tests, animals were killed. (a) Schedule of drug treatments and behavioral tests except MWM test for rats. (b) Schedule of drug treatments and MWM test for rats. LA: locomotor activity. OR: object recognition. MWM: Morris water maze. SAC: sacrifice.

This was performed as described previously [23] with minor modifications. On day 21 (Figure 1(a)), after the stress rats were placed individually in a black wooden box (80 × 80 × 40 cm) which was with the floor divided into four identical squares. Line crossings (with all four paws placed into a new square) and rears (with both front paws raised from the floor) were recorded in a 5-min period.

#### 2.5.2. Object Recognition Test

The test was carried out as described previously [23]. On day 21 (Figure 1(a)), each rat was allowed to move freely in a black wooden box for 5 min as habituation; locomotor activity was simultaneously recorded (see locomotor activity test part). Twenty-four hours later, rats were individually placed in the center of the box containing two identical objects (Lego blocks) located in the two diagonal corners. The cumulative time spent in exploring each object (object A and object B) was recorded separately during 5-min period.

Exploration was defined as touching or smelling or being close to (within 2 cm) the object. On day 23, each rat was tested for memory using the same procedure except that one of the familiar objects was replaced with a totally novel object (object C replacing object B). The cumulative time spent in exploring each object was recorded, respectively, during 5-min period. Formula RI = *T*2/(*T*1 + *T*2) was used to calculate the recognition index (RI). *T*2 is the cumulative time in exploring object B or object C. *T*1 is the cumulative time in exploring object A.

#### 2.5.3. Morris Water Maze Test

The role of XYS in memory was further confirmed using the Morris water maze test. This test was carried out as described previously [23]. The apparatus consisted of a circular, plastic pool (174 cm diameter, 72.5 cm high) located in a well-illuminated room with external cues visible from the inside of the pool, which was filled with opaque water (22–24°C). On day 21 (Figure 1(b)), a visible circular platform (10 cm diameter) was 2 cm higher than the water in one of the four quadrants. Rats were trained to escape by swimming to the platform. On day 22, a hidden circular platform (10 cm diameter) was submerged 2 cm under the water in one of four quadrants. The acquisition trials (training to escape to the hidden platform) were carried out for three consecutive days (6 trials for 2 days and 4 trials for 1 day) (Figure 1(b)). The latency (the time taken to climb onto the platform) for each rat was recorded and the cut-off time is 90 sec. On day 25, the probe trial was performed with the platform removed to assess spatial memory. Rat was allowed to swim for 90 sec. The numbers of entries into the target quadrant and time spent in the target quadrant where the platform was previously located were recorded.

### 2.6. Western Blotting

Hippocampal tissues were processed and Western blot analysis was performed as described previously [24]. Samples were separated using SDS-PAGE and then transferred to PVDF membranes. Sample loading quantility for PSD-95 is 10 *μ*g and that for SYP is 3 *μ*g. Membranes were incubated with Mouse Anti-PSD-95 (1 : 500) or Mouse Anti-Synaptophysin (1 : 50000). Both of antibodies were purchased from BD Transduction Laboratories. Beta-actin (1 : 1000) was purchased from Sigma. Membranes were incubated overnight with primary antibodies. After washing, the membranes were incubated with goat anti-mouse secondary antibody (1 : 10000, Li-COR) for 1 h. The detection and quantification of specific bands were carried out using a fluorescence scanner (Odyssey Infrared Imaging System, LI-COR Biotechnology) at 800 nm wavelength.

### 2.7. Statistical Analysis

Data shown were expressed as mean value ± standard deviation (SD) and analyzed using one-way ANOVA except for the data of the body weight and acquisition training of the Morris water maze, which were analyzed by two-way ANOVA. Newman-Keuls tests were used for post hoc multiple treatment comparisons. Statistical significance was considered when *P* < 0.05.

## 3. Results

### 3.1. Effects of XYS on Body Weights and Locomotor Activity Test

**Figure 2:**
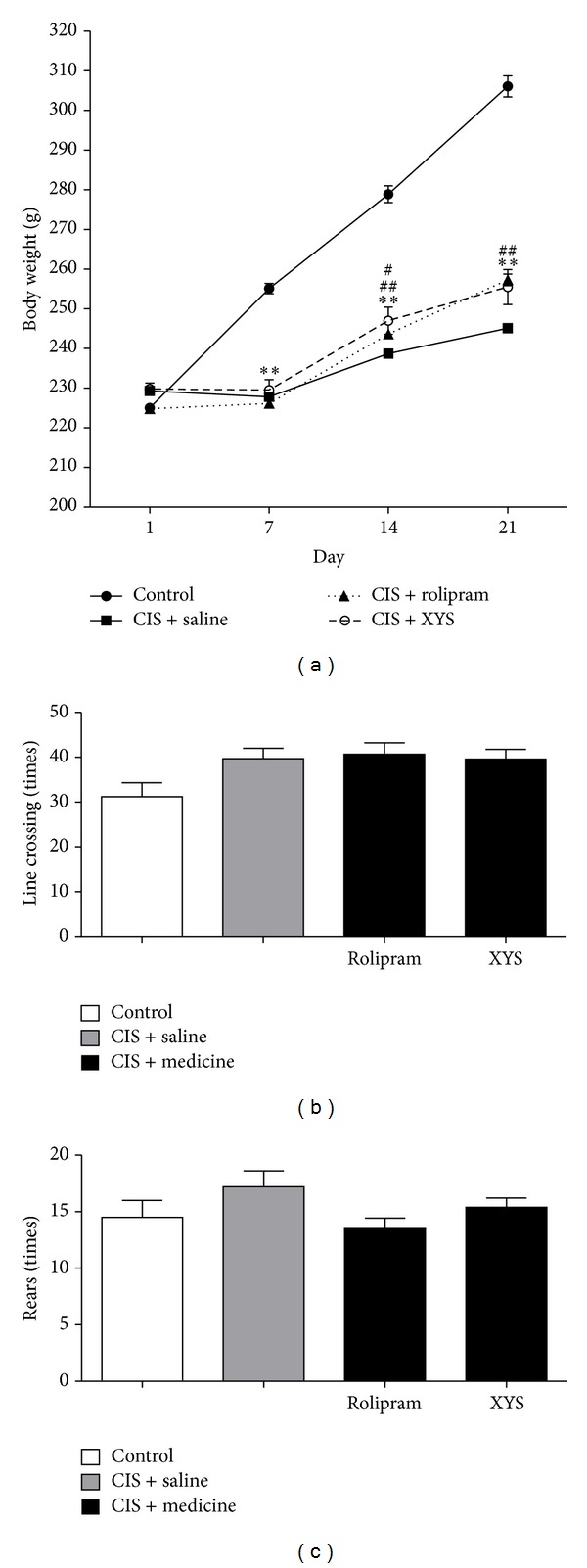
Effects of XYS on body weights and locomotor activity test. (a) After 1-week treatment, body weight gain of the CIS + saline group, and the CIS + rolipram group, the CIS + XYS group decreased markedly compared with that of the control group (*P* < 0.01). After 2-week treatment, body weight gain of the CIS + saline group, the CIS + rolipram group, and the CIS + XYS group increased compared with themselves after 1-week treatment. These differences were not significant. After 2-week treatment, body weight gain of the CIS + saline group, the CIS + rolipram group, and the CIS + XYS group decreased markedly compared with that of the control group (*P* < 0.01). Body weight gain of the CIS + XYS group increased compared with that of the CIS + saline group significantly (*P* < 0.05). After 3-week treatment, body weight gain of the CIS + saline group, the CIS + rolipram group, and the CIS + XYS group decreased markedly compared with that of the control group (*P* < 0.01). Body weight gain of the CIS + rolipram group and the CIS + XYS group increased compared with the CIS + saline group significantly (*P* < 0.05). (b) There is no significant difference between any two groups in four groups in locomotor activity, as assessed by line crossings and rears after stress on day 21. Values shown are means ± SD of ten rats per group. **P < 0.01 versus the control group. ^#^ *P* < 0.05, ^##^ *P* < 0.01 versus the CIS + saline group.

In this experiment, body weights of rats were monitored throughout 21 days to evaluate the potential effect of XYS. Body weight gain in all groups except the control group tended to be decreased statistically from the control group through whole 21 days. After 2-week and 3-week treatments, XYS and rolipram led to a slower gain of body weights. (Figure 2(a)) The treatment did not display significant changes in locomotor activity (Figures 2(b) and 2(c)).

### 3.2. Effects of XYS on Object Recognition Test

**Figure 3:**
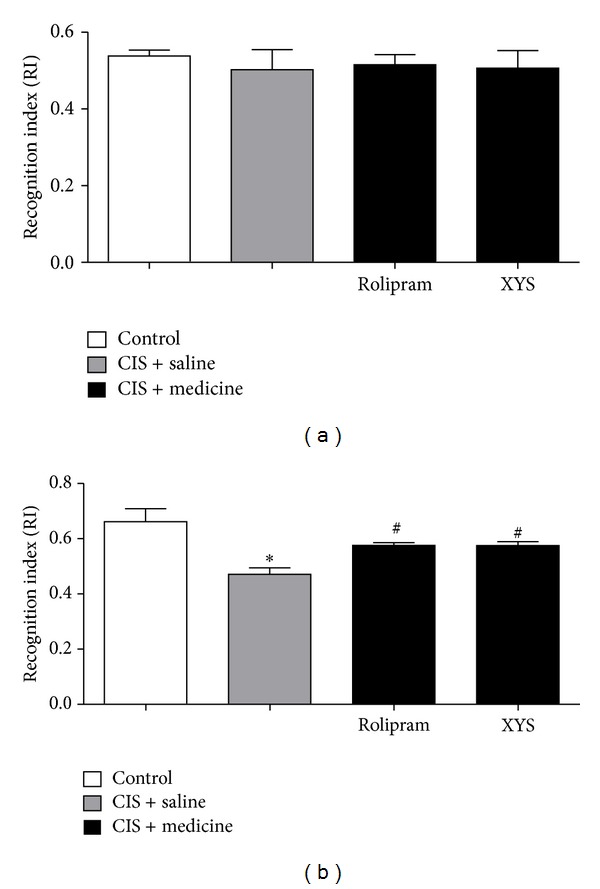
Effects of XYS on object recognition test. (a) There was no significant difference in RI between any two groups in the control group, the CIS + saline group, the CIS + rolipram group, and the CIS + XYS group when object A and object B were identical. (b) When object A was different from object C, RI of the CIS + saline group was lower than that of the control group significantly (*P* < 0.05). RIs of the CIS + rolipram group and the CIS + XYS group were higher than these of the CIS + saline group significantly (*P* < 0.05). Values shown are means ± SD of ten rats per group. *P < 0.05 versus the control group. ^#^ *P* < 0.05 versus the CIS + saline group.

In this experiment, rats in the CIS + saline group displayed worse recognition index in object recognition test compared with the control group. Rolipram or XYS treatments increased the recognition index compared with the CIS + saline group (Figure 3).

### 3.3. Effects of XYS on Morris Water Maze

**Figure 4:**
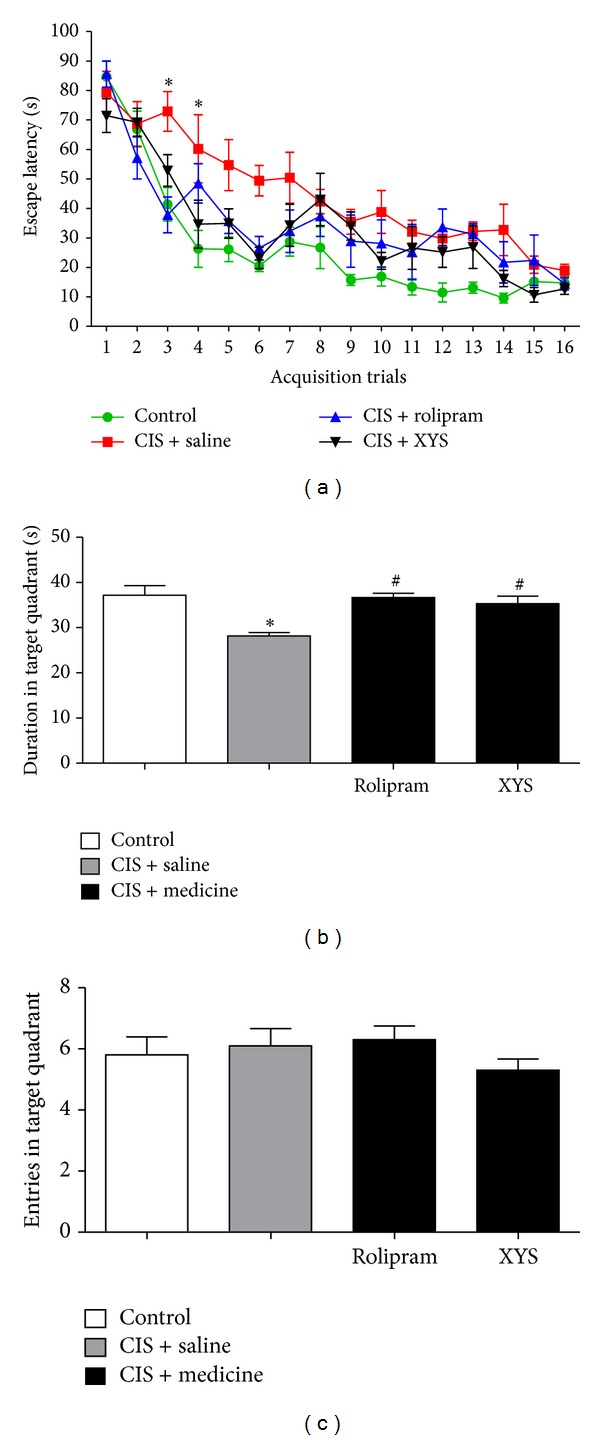
Effects of XYS on Morris water maze. (a) During the 3-day acquisition training, all the rats displayed progressive decreases in the escape latency to reach the hidden platform. Overall statistic compared by two-way ANOVA did not reveal significant changes, except in the 3rd and 4th acquisition trials (*P* < 0.05). (b) In the probe trial test performed 24 h after the last acquisition trial, CIS + saline group displayed significant decrease compared with the control group (*P* < 0.05) in duration in target quadrant. CIS + rolipram group and CIS + XYS group displayed significant increase compared with CIS + saline group (*P* < 0.05) in duration in target quadrant. (c) As entries in target quadrant, there are no significant changes in groups. Values shown are means ± SD of ten rats per group. *P < 0.05 versus the control group. ^#^ *P* < 0.05 versus the CIS + saline group.

In the experiment, rats displayed progressive decreases in the escape latency to reach the hidden platform during the 3-day acquisition training, especially in the 3rd and 4th acquisition trials for the CIS + saline group (Figure 4(a)). CIS + saline group displayed significant decrease compared with the control group in duration in target quadrant. CIS + rolipram group and CIS + XYS group displayed significant increase compared with the CIS + saline group in duration in target quadrant (Figure 4(b)). As entries in target quadrant, there are no significant changes in groups (Figure 4(c)).

### 3.4. Effect of XYS on PSD-95, SYP in the Hippocampus

**Figure 5:**
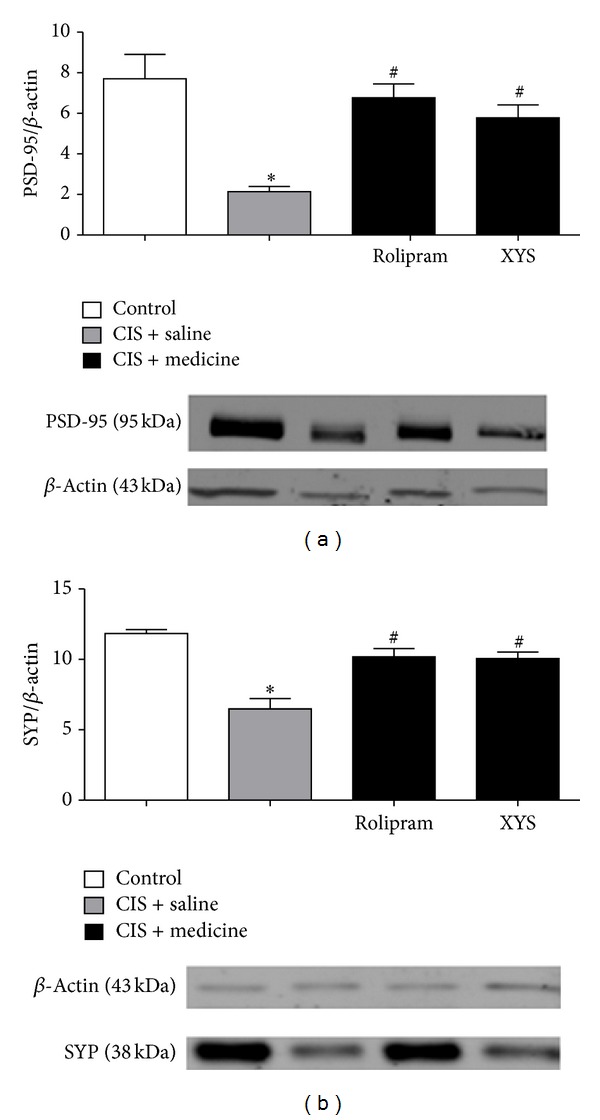
Effect of XYS on PSD-95, SYP in the hippocampus. Changes in expression of PSD-95 (a) or SYP (b) in the hippocampus. Bottom panels were representative immunoblots of PSD-95 or SYP detected by Western blotting. Top panels were the corresponding quantification. (a) PSD-95 in the CIS + saline group displayed decrease significantly in the hippocampus compared with that in the control group (*P* < 0.05). PSD-95 in the CIS + rolipram group and CIS + XYS group displayed increase significantly compared with that in the CIS + saline group (*P* < 0.05). (b) SYP in the CIS + saline group displayed decrease significantly in the hippocampus compared with that in the control group (*P* < 0.05). SYP in the CIS + rolipram group and CIS + XYS group displayed increase significantly compared with that in the CIS + saline group (*P* < 0.05). Values shown are means ± SD of ten rats per group. Values shown are means ± SD of ten rats per group. *P < 0.05 versus the control group. ^#^ *P* < 0.05 versus the CIS + saline group.

In this experiment, Western blotting results indicated that the CIS + saline group displayed decrease significantly in PSD-95 (Figure 5(a)) and SYP (Figure 5(b)) in the hippocampus compared with the control group. The CIS + rolipram group and CIS + XYS group displayed significant increase in PSD-95 (Figure 5(a)) and SYP (Figure 5(b)) in the hippocampus compared with the CIS + saline group (Figure 5).

## 4. Discussion

The purpose of this experiment was to dig out the mechanism of XYS on CIS induced learning and memory deficit in rats.

In TCM, the syndrome of stagnation of liver qi can lead to poor digestion and weight loss [25]. CIS can slow weight gain of rats; especially CIS was given in the morning [26, 27]. Symptoms of LSSDS include poor digestion, weight loss, diarrhea, bad appetite, and so on. Stress exposure 3 hours per day for 21 days leads to LSSDS, XYS decoction can soothe the liver and strengthen the spleen, and our previous study suggests that leptin receptor (ob-R) in the arcuate nucleus may act as the target of XYS in regulating the symptoms such as appetite and bodyweight loss under chronic stress with LSSDS [19]. Normal function of the liver and the spleen plays a beneficial role in turning the foods and drinks into nutrition. Nutrition can not only keep physical normality such as regular weight gain but also keep mental health such as memory and learning.

CIS leads to learning and memory deficit in object recognition test [28]. Chronic stress prolongs the latent period and decreases the times crossing the target quadrant in Morris water maze [29]. Chronic multiple stress can prolong the escape latency and duration of exploring in the target quadrant [30]. The memory enhancing effect of XYS is verified in Morris water maze task [31, 32].

Synaptic plasticity is the important part of neurobiology for learning and memory ability [33]. Synapse is the key to neuron networking and information transmission [34]. Reduction of neuronal synaptic plasticity and function deficit do harm to the ability of learning and memory [35]. PSD-95 is the postsynaptic membrane marker and SYP is the presynaptic membrane marker [36].

PSD-95 knocked out mice or PSD-95-mutant mice have LTP and learning and memory deficit [37, 38]. Chronic restraint stress leads to PSD-95 decreases in hippocampal CA3 areas of rats and the stress leads to the impairment of learning and memory ability [39]. Analog 165 can obviously improve the learning and memory ability and also increase the expression of PSD-95 [40]. Environmental enrichment significantly improves the memory damage induced by cerebral ischemia and upregulated the expression of PSD-95 mRNA in the ipsilateral cortex and hippocampus of occluding the right middle cerebral artery rats [41].

Immobilization stress exposure decreases expression of SYP in the hippocampus [42]. Patients with Alzheimer's disease were found that their PSD-95 and SYP decrease in the hippocampus [43, 44]. Cognitive decline of patients with Alzheimer's disease correlates with changes in the hippocampus and the content of cortical presynaptic vesicle protein SYP content [45, 46].

The above data suggested that XYS could improve the situation of slow weight gain induced by CIS, improve the ability of learning and memory, reverse the symptom of liver stagnation and spleen deficiency syndrome (LSSDS) in rats, and increase the levels of PSD-95 and SYP on the hippocampal nerve synapses to improve synaptic plasticity. Several groups of bioactive nature products have been identified from the herbs in the XYS decoction as cited by our previous published paper [18], combined with our previously reported data that XYS decoction ameliorates impairment of cellular plasticity and resilience underlying pathophysiology of mood disorders [18], and there were some inclusions appearing that low-dose XYS (1.927 g/kg/d) decoction containing serum decreases apoptotic rate of neuron significantly *in vitro *and XYS decoction (3.854 g/kg/d) improved chronic immobilization stress induced learning and memory deficit *in vivo *[20].

Further studies will be focused on the relationship between different doses of XYS decoction serum concentration and improvement of learning and memory deficit induced by CIS in rats.

## References

[1] Ballerini P, Di Iorio P, Caciagli F (2006). P2Y2 receptor up-regulation induced by guanosine or UTP in rat brain cultured astrocytes. *International Journal of Immunopathology and Pharmacology*.

[2] Zhu W, Mantione KJ, Casares FM, Sheehan MH, Kream RM, Stefano GB (2006). Cholinergic regulation of endogenous morphine release from lobster nerve cord. *Medical Science Monitor*.

[3] Martin SJ, Grimwood PD, Morris RGM (2000). Synaptic plasticity and memory: an evaluation of the hypothesis. *Annual Review of Neuroscience*.

[4] Kim JJ, Diamond DM (2002). The stressed hippocampus, synaptic plasticity and lost memories. *Nature Reviews Neuroscience*.

[5] Lee T, Jarome T, Li SJ, Kim JJ, Helmstetter FJ (2009). Chronic stress selectively reduces hippocampal volume in rats: a longitudinal magnetic resonance imaging study. *NeuroReport*.

[6] Srikumar BN, Raju TR, Rao BSS (2007). Contrasting effects of bromocriptine on learning of a partially baited radial arm maze task in the presence and absence of restraint stress. *Psychopharmacology*.

[7] Radecki DT, Brown LM, Martinez J, Teyler TJ (2005). BDNF protects against stress-induced impairments in spatial learning and memory and LTP. *Hippocampus*.

[8] McLaughlin KJ, Gomez JL, Baran SE, Conrad CD (2007). The effects of chronic stress on hippocampal morphology and function: an evaluation of chronic restraint paradigms. *Brain Research*.

[9] Ramkumar K, Srikumar BN, Rao BSS, Raju TR (2008). Self-stimulation rewarding experience restores stress-induced CA3 dendritic atrophy, spatial memory deficits and alterations in the levels of neurotransmitters in the hippocampus. *Neurochemical Research*.

[10] Li XH, Chen JX, Yue GX (2013). Gene expression profile of the hippocampus of rats subjected to chronic immobilization stress. *PLoS ONE*.

[11] Wang HN, Jin X, Zheng H (2008). The effect of different intensity and duration of chronic immobilization stress on the ability of special learning and memory in rats. *Chinese Journal of Behavioral Medical Science*.

[12] Li W, Chen JX, Yang JX (2003). The effect of compounds of soothing liver, invigorating spleen, tonifying kidney on the praxiology and immunological function of chronic immobilization stressed rats. *Acta Laboratorium Animalis Scientia Sinica*.

[13] Li MT, Xiang H (2010). Advances in effective ingredients and pharmacological action of Xiaoyao Pill or Xiaoyao San research. *Journal of Chinese Medicinal Materials*.

[14] Chen JX, Ji B, Lu Z, Hu LS (2005). Effects of Chai Hu (Radix Burpleuri) containing formulation on plasma *β*-endorphin, epinephrine and dopamine in patients. *The American Journal of Chinese Medicine*.

[15] Zhao H, Wan X, Chen J (2009). A mini review of traditional Chinese medicine for the treatment of depression in China. *The American Journal of Chinese Medicine*.

[16] Li W, Chen JX (2005). Changes of BDNF TrkB NT3 in hippocampus of rats of chronic immobilization stress model and effect of Xiaoyaosan. *Chinese Archives of Traditional Chinese Medicine*.

[17] Zhu WF, He QH (2003). *The Modern TCM Clinical Diagnostics*.

[18] Chen JX, Li W, Zhao X, Yang JX (2008). Effects of the Chinese traditional prescription Xiaoyaosan decoction on chronic immobilization stress-induced changes in behavior and brain BDNF, TrkB, and NT-3 in rats. *Cellular and Molecular Neurobiology*.

[19] Wang SX, Chen JX, Yue GX, Bai MH, Kou MJ, Jin ZY (2012). Xiaoyaosan decoction regulates changes in neuropeptide Y and leptin receptor in the rat arcuate nucleus after chronic immobilization stress. *Evidence-Based Complementary and Alternative Medicine*.

[20] Meng ZZ, Hu JH, Chen JX, Yue GX (2012). Xiaoyaosan decoction, a traditional Chinese medicine, inhibits oxidative-stress-induced hippocampus neuron apoptosis in vitro. *Evidence-Based Complementary and Alternative Medicine*.

[21] Andrus BM, Blizinsky K, Vedell PT (2012). Gene expression patterns in the hippocampus and amygdala of endogenous depression and chronic stress models. *Molecular Psychiatry*.

[22] Thome J, Pesold B, Baader M (2001). Stress differentially regulates synaptophysin and synaptotagmin expression in hippocampus. *Biological Psychiatry*.

[23] Li YF, Cheng YF, Huang Y (2011). Phosphodiesterase-4D knock-out and RNA interference-mediated knock-down enhance memory and increase hippocampal neurogenesis via increased cAMP signaling. *Journal of Neuroscience*.

[24] Carlin RK, Grab DJ, Cohen RS, Siekevitz P (1980). Isolation and characterization of postsynaptic densities from various brain regions: enrichment of different types of postsynaptic densities. *Journal of Cell Biology*.

[25] Yan C, Zhang ZC, Deng ZY (1995). Clinical and experimental study of immunological mechanism of ‘liver bears the dispersive effect’. *Chinese Journal of Basic Medicine in Traditional Chinese Medicine*.

[26] Andrus BM, Blizinsky K, Vedell PT (2012). Gene expression patterns in the hippocampus and amygdala of endogenous depression and chronic stress models. *Molecular Psychiatry*.

[27] Rybkin II, Zhou Y, Volaufova J, Smagin GN, Ryan DH, Harris RBS (1997). Effect of restraint stress on food intake and body weight is determined by time of day. *The American Journal of Physiology—Regulatory Integrative and Comparative Physiology*.

[28] Beck KD, Luine VN (1999). Food deprivation modulates chronic stress effects on object recognition in male rats: role of monoamines and amino acids. *Brain Research*.

[29] Cai YY, Zhang WG, Shi SX (2008). Effects of chronic stress on learning ability and expression of neuronal cell adhesion molecule in hippocampus in rats. *Shanghai Archives of Psychiatry*.

[30] Zheng G, Luo WJ, Chen YM, Liu MC, Ma JL, Chen JY (2011). Effects of chronic multiple stress on learning and memory and the expression and phosphorylation of cerebral ERK of rats. *Chinese Journal of Applied Physiology*.

[31] Morris RGM, Garrud P, Rawlins JNP, O’Keefe J (1982). Place navigation impaired in rats with hippocampal lesions. *Nature*.

[32] Remodes M, Schuman EM (2004). Role for a cortical input to hippocampal area CA1 in the consolidation of a long-tem memory. *Nature*.

[33] Zhu CG (2002). *Neuroanatomy*.

[34] Kim JH, Park YK, Kim JH, Kwon TH, Chung HS (2006). Transient recovery of synaptic transmission is related to rapid energy depletion during hypoxia. *Neuroscience Letters*.

[35] Coleman P, Federoff H, Kurlan R (2004). A focus on the synapse for neuroprotection in Alzheimer disease and other dementias. *Neurology*.

[36] Oliveira TG, Chan RB, Tian H (2010). Phospholipase D2 ablation ameliorates Alzheimer’s disease-linked synaptic dysfunction and cognitive deficits. *Journal of Neuroscience*.

[37] Kennedy MB (2000). Signal-processing machines at the postsynaptic density. *Science*.

[38] Migaud M, Charlesworth P, Dempster M (1998). Enhanced long-term potentiation and impaired learning in mice with mutant postsynaptic density-95 protein. *Nature*.

[39] Liao M, Liu NB, Zhang MH (2003). The change of synapsin and PSD 95 expression in the hippocampal neurons of rats with learning and memory impairment by chronic restraint stress. *Journal of Huazhong University of Science and Technology ( Health Science Edition)*.

[40] Lu S, Lei YP, Wang PW, Wang R, Sheng SL, Tian JZ (2007). The effects of analog 165 of APP 5-mer peptide on praxiology, expression of PSD95 and Shank 1 in Alzheimer disease rats. *Chinese Journal Neuroimmunology and Neurology*.

[41] Ye LJ, Xu XH, Wang YM (2008). The effects of enriched environment on structural modification of synaptic interface and PSD-95 mRNA of rats after transient focal cerebral ischemia. *Acta Psychologica Sinica*.

[42] Thome J, Pesold B, Baader M (2001). Stress differentially regulates synaptophysin and synaptotagmin expression in hippocampus. *Biological Psychiatry*.

[43] Terry RD, Masliah E, Salmon DP (1991). Physical basis of cognitive alterations in Alzheimer’s disease: synapse loss is the major correlate of cognitive impairment. *Annals of Neurology*.

[44] Sze C, Troncoso JC, Kawas C, Mouton P, Price DL, Martin LJ (1997). Loss of the presynaptic vesicle protein synaptophysin in hippocampus correlates with cognitive decline in Alzheimer disease. *Journal of Neuropathology and Experimental Neurology*.

[45] Antonova I, Arancio O, Trillat A (2001). Rapid increase in clusters of presynaptic proteins at onset of long-lasting potentiation. *Science*.

[46] Siew LK, Love S, Dawbarn D, Wilcock GK, Allen SJ (2004). Measurement of pre- and post-synaptic proteins in cerebral cortex: effects of post-mortem delay. *Journal of Neuroscience Methods*.

